# Beyond Conventional Control: Insights Into Drug-Resistant Hypertension

**DOI:** 10.7759/cureus.43617

**Published:** 2023-08-17

**Authors:** Pratyaksh Chhabra, Rajoshee R Dutta, Prerna Sahu, Abhishek Joshi

**Affiliations:** 1 Medicine and Surgery, Jawaharlal Nehru Medical College, Datta Meghe Institute of Higher Education and Research, Wardha, IND; 2 Community Medicine, Jawaharlal Nehru Medical College, Datta Meghe Institute of Higher Education and Research, Wardha, IND

**Keywords:** sympathetic activation, management, diagnosis, pathogenesis, drug-resistant hypertension

## Abstract

It is believed that 9-18% of patients with hypertension have resistant hypertension, a serious medical disease. The increased cardiovascular risk associated with this illness demands appropriate diagnosis and treatment. It is necessary to conduct an in-depth investigation of the various etiologies, indicators of risk, and multiple disorders of resistant hypertension. This is crucial in order to establish the diagnosis and make the best decisions regarding therapy. Treatment should also take lifestyle changes into account in addition to medicinal and interventional therapy. When there is a suspicion of resistant hypertension, examining the medications used to treat the hypertensive patient after ruling out pseudo hypertension, improper blood pressure monitoring and control, and the white-coat effect are necessary. Resistant hypertension, according to a specific definition, is a condition that cannot be treated with more than two antihypertensive drugs, including a diuretic. An effective multidrug therapy for the treatment of resistant hypertension includes angiotensin-converting enzyme inhibitors, angiotensin II receptor blockers, beta-blockers, diuretics, long-acting calcium channel blockers, and mineralocorticoid receptor antagonists. However, alternative, cutting-edge treatments, such as renal denervation or baroreflex activation, could develop a brand-new avenue for decreasing blood pressure. These new surgical interventions might prove out to be of immense importance in coming times. Secondary causes of resistant hypertension, such as obstructive sleep apnea, coronary artery diseases, nephropathy, or endocrinal diseases, must be checked out in order to make an accurate diagnosis of this illness. This review article briefly summarizes the epidemiology, risk factors, causes, pathogenesis, diagnosis, and treatment approaches that may help with the long-term management of resistant hypertension.

## Introduction and background

Hypertension is a chronic and highly complex disease. Of all the cardiovascular risk factors, hypertension has the highest prevalence, at 30-45%. One of the primary ways to ensure increased patient quality of life over the long run is to obtain blood pressure readings below the target blood pressure (usually below 140/90 mmHg). Extreme phenotypes of hypertension, particularly drug-resistant hypertension or resistant hypertension, exist. Genetic factors might be of importance too. Several genes have been linked to failure to respond to antihypertensive medicine. Epigenetics is vital because environmental aspects play a part in the evolution of resistant hypertension [1]. Many pathological and physiological processes are associated to an increased risk of the cardiovascular system leading to resistant hypertension because of the heightened invigoration of the renin-angiotensin system and synthesis of corticosteroids. In addition to atherosclerotic disease and arterial stiffness, people with resistant hypertension typically have both endothelial dysfunction and increased sympathetic nervous system activity.

Resistant hypertension is a condition in which a minimum of three antihypertensive drugs, a diuretic included, are not enough to bring down the ambulatory blood pressure to under the required levels [2]. Resistant hypertension comes in two forms: managed and uncontrolled. Despite taking the prescribed treatment and making the optimum lifestyle adjustments, individuals with uncurbed resistant hypertension cannot decrease their blood pressure to a target blood pressure (usually below 140/90 mmHg). According to estimates, 8-13% of people on antihypertensive medications are affected [3]. Despite the pharmaceutical inhibition of the sympathetic nervous system that is currently accessible, roughly 50% of patients exhibit inadequate regulation, and in routine clinical practice, pharmacotherapy does not produce sufficient results. Unidentified secondary causes of hypertension and a patient's or doctor's lack of compliance are the most frequent reasons for therapeutic failure. It can be related to resistant hypertension in roughly 10% of instances, which is brought on by the overactive sympathetic nervous system [4].

## Review

Methodology

**Figure 1:**
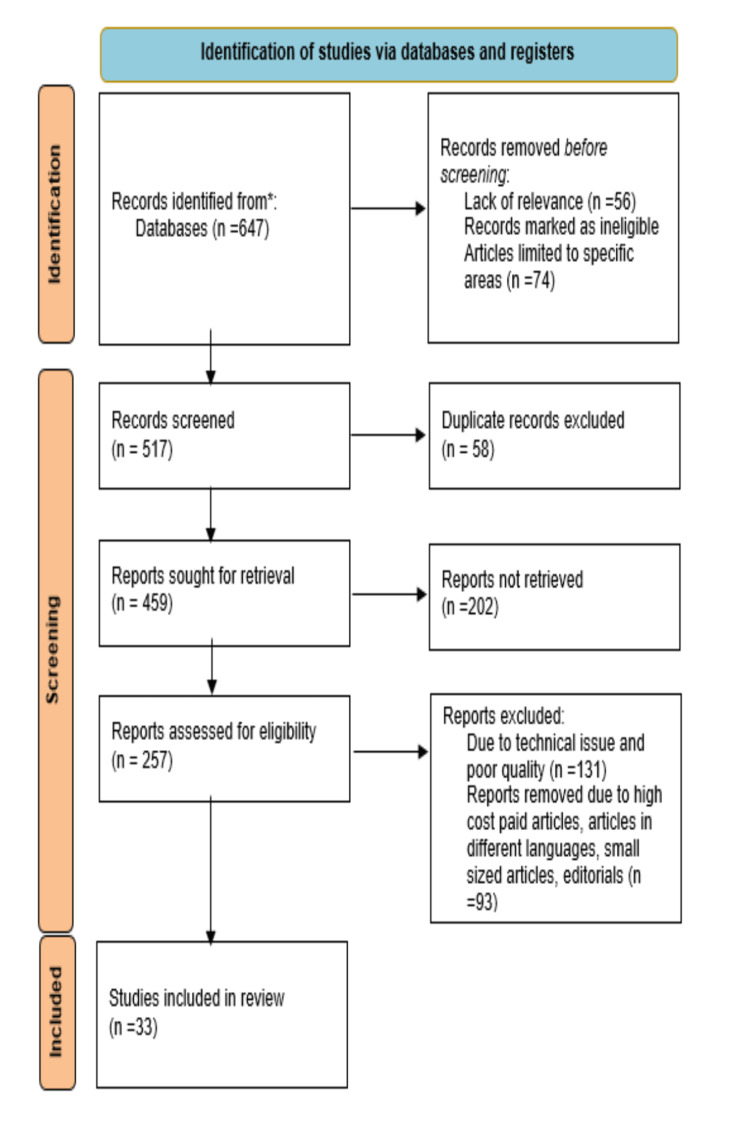
PRISMA methodology PRISMA: Preferred Reporting Items for Systematic Reviews and Meta-Analyses

Using the electronic databases PubMed, MEDLINE, Embase, Google Scholar, and ResearchGate, a search of the English-language literature was done. It was also the subject of a different search. The query terms were "treatment resistant hypertension" OR "drug resistant hypertension"; "etiology" OR "causes; "pathogenesis," OR "pathophysiology”; “diagnosis” OR “investigations”; "treatment” OR "modalities.” The articles in this review meet the following requirements: Studies conducted exclusively on hypertension, drug-resistant hypertension, and new treatment interventions are considered. Studies conducted in English over the preceding 10 years are also included. Figure 1 highlights the Preferred Reporting Items for Systematic Reviews and Meta-Analyses (PRISMA) method's use in research methodology.

Defining apparent, true, and pseudo-resistant hypertension

**Figure 2:**
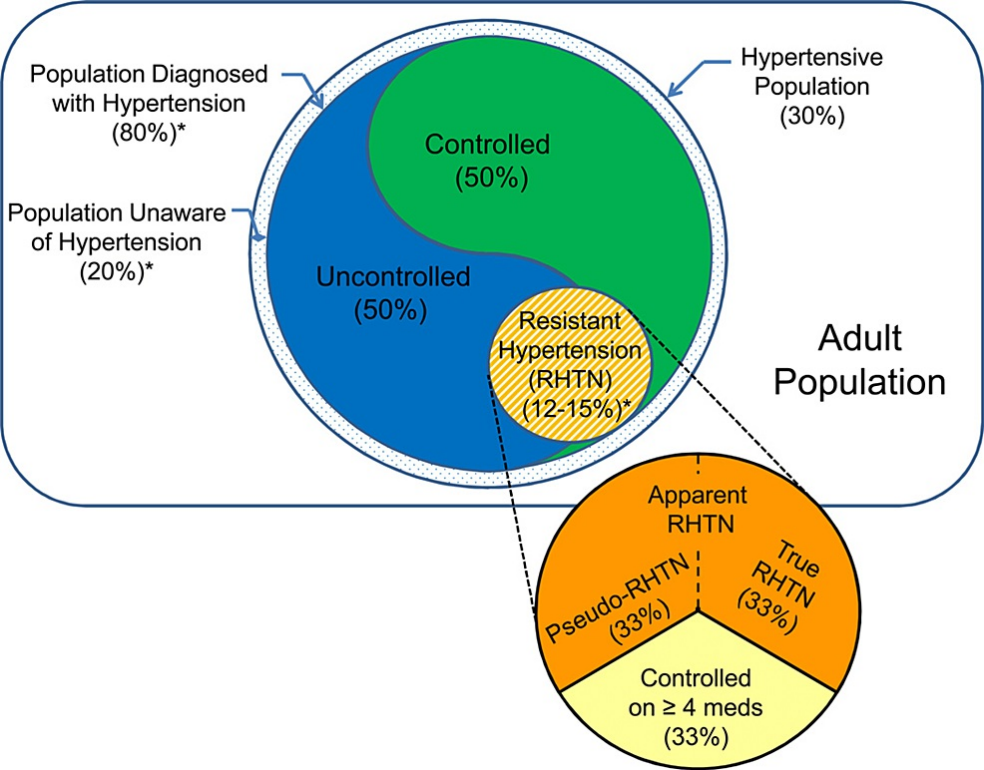
Venn diagram of the prevalence of resistant hypertension

To recognize a set of high-risk individuals who would get an advantage from specialized care, together with assessment and therapy of secondary reasons for hypertension, resistant hypertension was initially defined [3]. The definition was created in a scientific statement by the American Heart Association (AHA) as a blood pressure that stays beyond the target despite receiving the recommended dosages of three antihypertensive medications from various classes, one of which should preferably be a diuretic. In addition, a particular individual remains resistant if their blood pressure is controlled by a fourth antihypertensive drug. As a result, the group of resistant hypertensives consists of people whose hypertension is managed and controlled by ambulatory measurement (Figure 2) [5]. Resistant and pseudo-resistant hypertension are not differentiated in the AHA's definition of resistant hypertension. People with elevated office blood pressure brought on by white-coat hypertension, inaccurate blood pressure readings, or medication nonadherence do not have resistant hypertension; instead, they have pseudo-resistant hypertension. When pointing out a set of individuals with an ambulatory blood pressure >140/90 mmHg while taking three antihypertensive drugs, epidemiological research adopted the phrase apparent resistant hypertension to emphasise that pseudo-resistance had not been excluded [5].

Epidemiology

Epidemiological statistics show that antihypertensive medication effectiveness varies widely. In the National Health and Nutrition Examination Survey (NHANES) trial, only 53% of individuals taking antihypertensive medications met their target blood pressure values. Individuals with diabetes mellitus and chronic kidney ailment showed an even decreased percentage of blood pressure goal limits, i.e., 130/80 mmHg [5]. About 68,000 people were treated for hypertension in a comprehensive research based on the Spanish Ambulatory Pressure Registry; 12% of these patients were identified as having resistant hypertension. In 62.5% of the patients, ambulatory blood pressure measurement indicated natural resistance to treatment, and in the remaining 37.5%, the white-coat effect was responsible for the therapy's ineffectiveness.

The Antihypertensive and Lipid-Lowering Treatment to Prevent Heart Attack Trial (ALLHAT) results were analyzed, and it was discovered that 27% of patients were taking at least three antihypertensive drugs at the time the study came to an end and that 47% of patients experienced treatment resistance in the first year of follow-up [6].

Risk factors

The most crucial cardiovascular continuum modifiable variable is hypertension. A hyperactive sympathetic nervous system disorder puts the patient at a greater risk for cardiovascular diseases. Co-occurring cardiorenovascular diseases, such as failure of the heart, fibrillation in the atria, and chronic renal ailments, are frequently present in patients with resistant hypertension. Sympathetic activity has been demonstrated to cause cardiorenovascular disease on its own, according to research [7].

An increase in blood pressure of 20 mmHg systolic and 10 mmHg diastolic doubles the risk of cardiovascular disease. Patients with elevated systolic and regular or lowered diastolic blood pressure have an exceptionally susceptible for cardiovascular diseases. Individuals with diabetes mellitus and chronic renal ailments are a specific population at increased risk for cardiorenovascular diseases. The pervasiveness of resistant hypertension in individuals with chronic renal illness has been underestimated, and we are unaware of the true incidence of chronic renal disorder in individuals with resistant hypertension [6,7]. Resistance may be brought on by obesity and chronic renal disease. Additional risk factors, which are typically linked to resistant hypertension, contribute to its development and progression in a synergistic manner. A diagnosis of poorly regulated resistant hypertension is supported by target-organ impairment, such as retinopathy, chronic kidney disease, dementia, and ventricular hypertrophy [8].

Causes of resistant hypertension

Non-compliance

Non-compliance is a common factor in resistant hypertension. Patients may refuse to take their prescription due to cost; side effects; a health belief system, such as the notion that pharmaceuticals are hazardous; or as a consequence of a plot between the medical community and pharmaceutical industry. A particularly challenging situation is when a patient incorrectly links all symptoms to drug side effects [9].

Patient-Consumed Substances That Aggravate Hypertension 

Patients with hypertension are encouraged to limit their salt intake to 2-3 g per day, refrain from adding salt, and avoid meals with high sodium content. Licorice, which can be found in candies, tea, and some tonics, has mineralocorticoid effects that can lead to hypertension and hypokalemia. Oral contraceptives also cause hypertension. Sulindac is the only nonsteroidal anti-inflammatory drug (NSAID) that does not elevate blood pressure. The risk of hypertension is doubled, and the chance of non-lobar intracerebral bleeding is increased with heavy alcohol consumption (>three drinks per day) [10].

Therapeutic Inertia

There are various causes of therapeutic inertia. The additional health issues that people with hypertension may experience, such as diabetes, arthritis, gastrointestinal issues, and headaches, may attract the attention of busy doctors. The focus of clinic visit shifts when a patient has problems in other areas and hypertension may go unnoticed. In addition, patients and doctors may hesitate to increase antihypertensive treatments due to the false notion that white-coat hypertension is benign. Therapeutic inertia can be overcome simply by taking your blood pressure at each visit and having a rule that your antihypertensive therapy will be enhanced whenever your target blood pressure is not met [9,10].

Diagnostic Inertia

When a patient does not respond to the typical antihypertensive treatment, it is essential to consider whether the therapy is suitable for that patient and to look into the underlying physiology causing hypertension. "Diagnostic inertia" is the failure to consider the causes of patients' uncontrolled blood pressure [9,10].

Pathogenesis 

Hyperaldosteronism

A turning point in the study of resistant hypertension's pathophysiology and treatment was the recognition of aldosterone as a significant component of medication resistance. These intricate mechanisms entail considerably more than the aldosterone-caused increase in salt reabsorption [11]. The significance of aldosterone's direct vasoconstrictive action through the myocytes of the vessel wall and dissolution of homeostasis conditioning appropriate tension have been decisively proven by the findings of various recent research. As a result, inhibiting aldosterone activity may be a valuable strategy for treating resistant hypertension [10,11].

Obstructive Sleep Apnea

Hypertension affects 50-60% of individuals with obstructive sleep apnea syndrome, which is medically measured by the apnea/hypopnea index (AHI). Individuals with obstructive sleep apnea appear to have a complex etiology for hypertension. There are a number of potential causes, including increased sympathetic nervous system stress, excessive activity of the heart, increased blood vessel resistance, accumulation of fluid brought on by hypoxia of the tissues, and the result of a raised aldosterone level. These factors contribute to rising blood pressure [11].

Sympathetic Nervous System and Kidneys in the Development of Resistant Hypertension 

One of the main pathological mechanisms in developing resistant hypertension appears to be the sympathetic nervous system. Further research is needed to determine the relationship between obstructive sleep apnea syndrome, abdominal obesity, and high aldosterone levels in individuals with resistant hypertension. Nonetheless, it appears that the sympathetic nervous system's increased activity is the primary factor linking these conditions [12].

Adrenergic hyperstimulation of the kidneys is a crucial pathomechanism that helps resistant hypertension develop. Numerous kidney-derived mechanisms encourage the development of resistant hypertension, such as impaired pressure natriuresis (caused by parenchymal and renal artery-involved chronic kidney disorders), which, under normal circumstances, is in charge of renal sodium excretion [12].

Secondary Basis of Resistant Hypertension

Five to 10 percent of resistant hypertension instances are the result of secondary factors. Their prevalence rises with increasing age, especially in connection to hypertension brought on by renal artery atherosclerotic stenosis. Parenchymal kidney diseases, vascular hypertension, hyperparathyroidism, coarctation of the aorta, and intracranial tumors are a few of the less common reasons for secondary resistant hypertension. Less common causes include Cushing's syndrome, pheochromocytoma, thyroid gland dysfunction (hyper- and hypothyroidism), and hyperparathyroidism [13,14].

Diagnosis

Secondary factors, such as obstructive sleep apnea, nephropathy, or endocrinal diseases, must be ruled out to identify resistant hypertension. Clinical evaluation, laboratory tests, and diagnostic techniques to determine organ damage are all included in the diagnostic workup [14].

Medical History and Clinical Examination

It includes information on the onset, course, and duration of hypertension, the treatment's adherence, and previous medications' effects, including any adverse side effects. Medical history must include recent prescription use, including herbal remedies and over-the-counter (OTC) drugs. Obstructive sleep apnea is indicated by audible snoring, daytime drowsiness, and witnessed apnea. Renal artery stenosis is suspected in cases of worsening kidney function and a history of coronary atherosclerotic disease [13]. Diaphoresis and palpitations after labile hypertension point to a possible pheochromocytoma. During a physical examination, carotid, femoral, or abdominal bruits are signs of stenosis of the artery. Moon facies, stomach striae, and central obesity suggest Cushing's syndrome. Reduced femoral pulses and a differential in blood pressure between the arm and thigh raise suspicion of coarctation of the aorta and aortoiliac illness [14].

Biochemical Evaluation

The routine metabolic profile, such as blood urea nitrogen, potassium, chloride, sodium, bicarbonate, glucose, albumin/creatinine ratio, estimated glomerular filtration rate utilizing the Chronic Kidney Disease Epidemiology Collaboration equation, and urate level, should be included in the biochemical evaluation. Recognizing premature stages of chronic kidney disease, marked by slightly to reasonably diminished estimated glomerular filtration rate (eGFR) and serum creatinine values typically within the population-based reference intervals, requires reporting of the eGFR [15,16]. The ratio of aldosterone to renin is a helpful prognostic test for primary aldosteronism, even in continuous antihypertensive therapy, even though such a high ratio has a high negative predictive value and low specificity. Twenty-four-hour urine and plasma cortisol levels are two more analyses. Estimating dietary salt and potassium consumption can be done using 24-hour urine obtained while the patient consumes their regular diet. For individuals with whom pheochromocytoma is conjectured, 24-hour urinary metanephrines or plasma metanephrines are an efficient screening tool [15].

Diagnostic Methods

Resistant hypertension can develop for a variety of biological and behavioral reasons. Medicines, obesity or volume overload, high blood sugar (diabetes), advanced age, kidney disorders, aldosteronism, and obstructive sleep apnea are a few of them. Pheochromocytoma, Cushing's syndrome, thyroid conditions, and coarctation of the aorta are less common. When trying to diagnose a patient with resistant hypertension, it is crucial to consider their medical history, past compliance, accurate blood pressure readings, physical condition, biochemical investigations, and imaging, which is non-invasive. It is crucial that an individual's examination comprises 24-hour ambulatory blood pressure monitoring. High-risk individuals with ambulatory blood pressure monitoring, like those with obstructive sleep apnea and chronic kidney disease, frequently have a "non-dipper" rhythm [15,16].

The following suggestions for a diagnostic strategy for resistant hypertension were made by the French Society of Hypertension in order to ensure accurate blood pressure readings: (A) Standardized equipment and the right cuff size should be utilized [15]. (B) Ambulatory or at-home blood pressure readings should be used to counteract the white-coat effect. Home blood pressure readings of less than 135/85 mmHg, 24-hour ambulatory readings of less than 130/80 mmHg, daytime ambulatory readings of less than 135/85 mmHg, and nighttime ambulatory readings of less than 120/70 mmHg are thresholds for uncontrolled hypertension [15]. (C) Whether the best triple-drug treatment is given should be determined [15]. (D) During drug analysis and pill counting, poor compliance of patients should be evaluated using a questionnaire [15]. (E) It is advised to look for conditions that could contribute to resistance to treatment (e.g., obesity, immoderate consumption of sodium in the diet, alcohol use, and drug interactions). If resistant hypertension is determined to be the cause, a hypertension expert should be consulted. Once real resistant hypertension has been established, the underlying cause should be determined, and end-organ damage and cardiovascular risk should be evaluated [15].

Treatment strategies

The optimal therapeutic alternatives, including pharmacological and interventional therapies and lifestyle changes, are chosen by the treatment approach [17].

Lifestyle Modifications

Sedentary, overweight, smoking, or drinking individuals without exercise routines, consuming a high-sodium diet, and having unfavorable attitudes toward medication are examples of bad or unhealthy lifestyles. Over 60% of people with resistant hypertension (12% body mass index (BMI) >40 kg/m^2^) are overweight or obese. Diabetes, hypertension, overweight, and elevated levels of cholesterol are some of the main risk factors for death and disease in both men and women. In an analysis of external, professional, and metabolic variables causing mortality and impairments, the research claims that inadequate nutrition may be held responsible for around one in five fatalities and that nicotine use was responsible for more than seven million deaths [18,19]. In people with hypertension, appropriate food and lifestyle changes, quitting smoking, and weight loss are strongly advised. Reduced risk of cardiovascular diseases has been associated with higher levels of physical activity and diet. All forms of exercise have a positive impact on lowering the threat of cardiovascular system diseases and early mortality. Regardless if the increased physical activity was for leisure, work, or household purposes, the benefits were still evident [19].

Adherence

One important modifiable component in the management of hypertension is medication adherence. Adherence is multifaceted in nature. In order to increase adherence, blood pressure control therapies must be multifaceted and patient centered. One technique to better hypertension drug adherence and blood pressure management is a multidimensional approach that includes planning at the level of the suffering individual and medical treatment provider, organization, and system. Communication abilities with hypertension patients, information sharing, and therapeutic simplicity are crucial. It is advised to choose medications with 24-hour blood pressure management in combination with an everyday single dose. The low cost of antihypertensive medications is crucial, especially for people with limited resources. It is quite challenging to closely assess treatment compliance. The main issue is that the recommended regimens are not followed through consistently. Patients should be carefully questioned about their success in taking all of their prescribed dosages and any side effects and inconveniences associated with dosing. Members of the family will frequently offer more objective evaluations of the adherence of the patient. However, such feedback should typically be given during the patient's attendance. The most precise way is a direct observation of the therapy. However, it is not feasible for chronic disorders. Self-reporting and pillbox counting are practical techniques, but they can also be easily manipulated [20,21].

Multidrug Therapy

Therapy is difficult to integrate into routine therapeutic practice. To control blood pressure, it is crucial to select a mix of antihypertensive medications in set dosages. Some antihypertensive drugs have the advantage of reducing insulin resistance and inflammation while improving arterial elasticity as measured by central blood pressure and pulse wave velocity [21,22]. An antagonist of mineralocorticoid receptors (aldosterone antagonists), such as spironolactone, is a component of multidrug therapy. Despite having sexual side effects and the potential to cause hyperkalemia, both medications are useful for treating resistant hypertension, mostly in people with diabetes mellitus and chronic renal ailments. A beta-blocker or a centrally functioning antihypertensive medication shall be provided if side effects arise or in the event of a nonresponse [22].

Device Therapies

The sympathetic nervous system is a neglected yet crucial mechanism in the management of hypertension. Antiadrenergic medications often rank fourth or fifth in guidelines for hypertension in national and worldwide society, which is highly uncommon. Numerous techniques and gadgets target the sympathetic nervous system to safely and successfully control blood pressure in people with resistant hypertension. True resistant hypertension may be more easily controlled with the new gadget therapy. Continuous positive airway pressure (CPAP), baroreceptor activation treatment, and renal sympathetic denervation were created to halt the progression of cardiovascular ailment, the main reason of death worldwide [21].

Catheter-Based Renal Denervation

Energy is delivered to the renal nerves by renal sympathetic denervation, which helps regulate blood pressure. For sympathetic denervation, which necessitates the ablation of the renal sympathetic nerves, a catheter emitting radiofrequency is percutaneously inserted beyond the femur and into the lumen of the two renal arteries [22]. Renal sympathetic denervation causes moderately severe discomfort in the abdomen during energy delivery because it stimulates the renal sensory nerves before surgical removal. Opiates and sedatives must be used throughout the procedures to manage pain [21].

Renal sympathetic denervation is a risk-less procedure for lowering ambulatory blood pressure in individuals with resistant hypertension, according to numerous observational studies.

Utilizing specialized radiofrequency ablation catheters, the efficacy was proven in the Symplicity HTN-1, HTN-2, and EnligHTN-1 investigations [23]. Individuals with diabetes and/or chronic kidney disease have an overactive sympathetic nervous system, which leads to fluid retention, increases hypertension, and accelerates the loss of renal function. Renal sympathetic denervation has been shown to be associated with a stable kidney function in those patients [24]. 

Baroreceptor Activation Therapy

Therapies, including baropacing or baroreceptor stimulation, may be helpful for patients with hypertension who are unresponsive to medication. Baroreceptors in the brainstem respond to an elevation in carotid transmural pressure by suppressing sympathetic and invigorating parasympathetic centers [25]. Any rise in blood pressure will thus gradually return to its initial level. The majority of baropacing studies used office blood pressure as their only effectiveness criterion, but just a single study that evaluated the reaction over the course of 24 hours found that blood pressure had decreased by 8/5 mmHg after half a year [26].

Continuous Positive Airway Pressure

Nasal CPAP breathing is the preferred therapy for mild to severe obstructive sleep apnea. CPAP has been shown to have a range of effects on blood pressure levels, but in few patient subgroups, such as people with critical obstructive sleep apnea or/and resistant hypertension, more significant outcomes have been reported. Improving CPAP outcomes entails addressing a variety of aspects to ensure patients receive effective and comfortable therapy, such as patient education, proper equipment selection and fit, gradual acclimatisation, comfort and mask adjustments, and humidification [27].

Discussion

Hypertension is the most prevalent risk factor for cardiovascular diseases and mortality globally. Our lives must include physical activity because it is healthy and a low-cost preventive measure. Losing weight; eating an increased-fiber, reduced-fat, and reduced-salt diet; and drinking alcohol with restraint are all examples of lifestyle modifications [28]. Approximately 50% of individuals exhibit inadequate control despite the availability of pharmaceutical antihypertensive treatments. Patients with resistant hypertension frequently also have concomitant heart failure, atrial fibrillation, or chronic kidney disease. Patients with resistant hypertension frequently have hypertensive illness of the myocardium, blood vessels, brain (particularly dementia connected to vessels), and kidney [29].

Blood pressure readings taken in the mornings at the clinic are typically used for the diagnosis and management of hypertension. There is evidence to suggest that the sleeping blood pressure estimates events of the cardiovascular system more accurately than the awake or 24-hour blood pressure average [30]. It has been shown that renal denervation might offer an extra therapy choice to lower blood pressure in resistant hypertension individuals with obstructive sleep apnea. The relative effects in resistant hypertension demand better comprehension [31].

It is crucial to make the schedules for managing resistant hypertension as straightforward as feasible. It indicates managing all concurrent comorbidities and managing blood pressure. The diagnostic strategy for resistant hypertension places a lot of emphasis on the detection and treatment of multisite arterial disease [32]. Cost-wise, there is no doubt that treating the effects of hypertensive target organ damage is less expensive than controlling blood pressure well in resistant hypertension with medications and cutting-edge treatment with devices [33].

## Conclusions

Resistant hypertension prevalence is difficult to precisely estimate, but it is required because it is on the rise and is becoming a more significant clinical issue. Due to the elevated likelihood of developing cardiovascular issues, affected individuals require careful selection of the most appropriate antihypertensive medicine, taking into account the key physiology and pathology leading to the development of resistant hypertension. Further research is required to determine the connection between obstructive sleep apnea, elevated aldosterone levels, and the development of resistant hypertension To ascertain a longer period consequences of kidney degeneration and baroreceptor stimulation, prospective studies are also necessary.
